# Functional and genetic screening of acute myeloid leukemia associated with mediastinal germ cell tumor identifies MEK inhibitor as an active clinical agent

**DOI:** 10.1186/s13045-016-0258-1

**Published:** 2016-03-31

**Authors:** Jessica T. Leonard, Philipp W. Raess, Jennifer Dunlap, Brandon Hayes-Lattin, Jeffrey W. Tyner, Elie Traer

**Affiliations:** Division of Hematology and Oncology, Oregon Health & Science University, 3181 SW Sam Jackson Park Road, Portland, OR 97239 USA; Department of Pathology, Oregon Health & Science University, 3181 SW Sam Jackson Park Road, Portland, OR 97239 USA; Center for Hematologic Malignancies, Oregon Health and Science University, 3181 SW Sam Jackson Park Road, Portland, OR 97239 USA; Department of Cell, Developmental, and Cancer Biology, Portland, OR USA; Knight Cancer Institute, Oregon Health & Science University, 3181 SW Sam Jackson Park Road, Portland, OR 97239 USA

**Keywords:** Acute myeloid leukemia, NRAS, Trametinib, Germ cell neoplasm, High-throughput nucleotide sequencing

## Abstract

**Background:**

Hematologic malignancies arising in the setting of established germ cell tumors have been previously described and have a dismal prognosis. Identification of targetable mutations and pathway dysregulation through massively parallel sequencing and functional assays provides new approaches to disease management.

**Case Presentation:**

Herein, we report the case of a 23-year-old male who was diagnosed with a mediastinal germ cell tumor and subsequent acute myeloid leukemia. A shared clonal origin was demonstrated through identification of identical NRAS and TP53 somatic mutations in both malignancies. The patient’s leukemia was refractory to standard therapies with short interval relapse. Functional assays demonstrated the patient’s blasts to be sensitive to the mitogen-activated protein kinase kinase (MEK) inhibitor trametinib, correlating with the activating NRAS mutation. The patient experienced a sustained partial remission while on trametinib therapy but ultimately suffered relapse of the germ cell tumor. The leukemic clone remained stable and sensitive to trametinib at that time.

**Conclusions:**

This case highlights the potential power of combining genetic sequencing and in vitro functional assays with targeted therapies in the treatment of rare diseases.

**Electronic supplementary material:**

The online version of this article (doi:10.1186/s13045-016-0258-1) contains supplementary material, which is available to authorized users.

## Background

Mediastinal germ cell tumors (GCT) are capable of malignant transformation into tumors of endodermal, mesodermal, and ectodermal origin. With regard to hematologic malignancies, GCT are most frequently associated with acute myeloid leukemia (often with megakaryoblastic differentiation); but myelodysplastic syndromes, malignant histiocytosis, myeloproliferative neoplasms, and acute lymphoblastic leukemia have all been described [1–7]. Identification of shared cytogenetic abnormalities in the GCT and hematologic malignancy (most commonly isochromosome 12p) indicate a clonal relationship and common precursor [8].

The prognosis for GCT with a concurrent hematologic malignancy is poor, as the disease is usually refractory to conventional chemotherapy and patients are unable to attain a clinical remission [9]. For those that do attain a remission, relapse without consolidative allogeneic stem cell transplant is inevitable, and successful outcomes are limited to two cases in which patients underwent allogeneic hematopoietic stem cell transplant early in the first complete remission [1, 10]. Given these dismal outcomes, novel therapeutic approaches are needed.

Here, we report the case of a young man with metasynchronous presentation of a GCT and acute myeloid leukemia (AML). Following failure of conventional cytotoxic chemotherapy, a functional in vitro assay was used to identify a targeted therapeutic agent with activity against the leukemic blasts: trametinib, an inhibitor of mitogen-activated protein kinase kinase (MEK). This in vitro sensitivity correlated with the identification of a somatic *NRAS* missense mutation, which also predicts sensitivity to MEK inhibition. The patient was treated with trametinib for 6 months, during which time his leukemia remained stable. This case highlights the utility of combining mutational profiling with functional in vitro testing of the leukemic clone to identify effective personalized therapies for rare malignancies.

## Case presentation

**Fig. 1:**
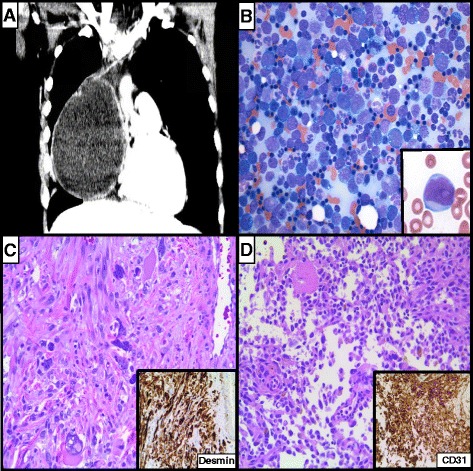
A 23-year-old male with a metasynchronous mediastinal germ cell tumor and acute myeloid leukemia. **a** Computed tomography demonstrated an 18-cm anterior mediastinal mass. **b** Bone marrow aspirate smear examination (4 months after germ cell tumor diagnosis) demonstrated acute erythroid leukemia 70–80 % markedly dysplastic and left-shifted erythroid precursors with myeloid blasts (*inset*) accounting for more than 20 % of non-erythroid cellularity. **c** Post-treatment resection of the mediastinal germ cell tumor demonstrated predominantly mature teratoma with focus of rhabdomyosarcoma (pictured) with desmin positivity (*inset*). **d** Separate focus of angiosarcoma was also identified in the mediastinal germ cell tumor (pictured), supported by CD31 positivity (*inset*)

**Fig. 2:**
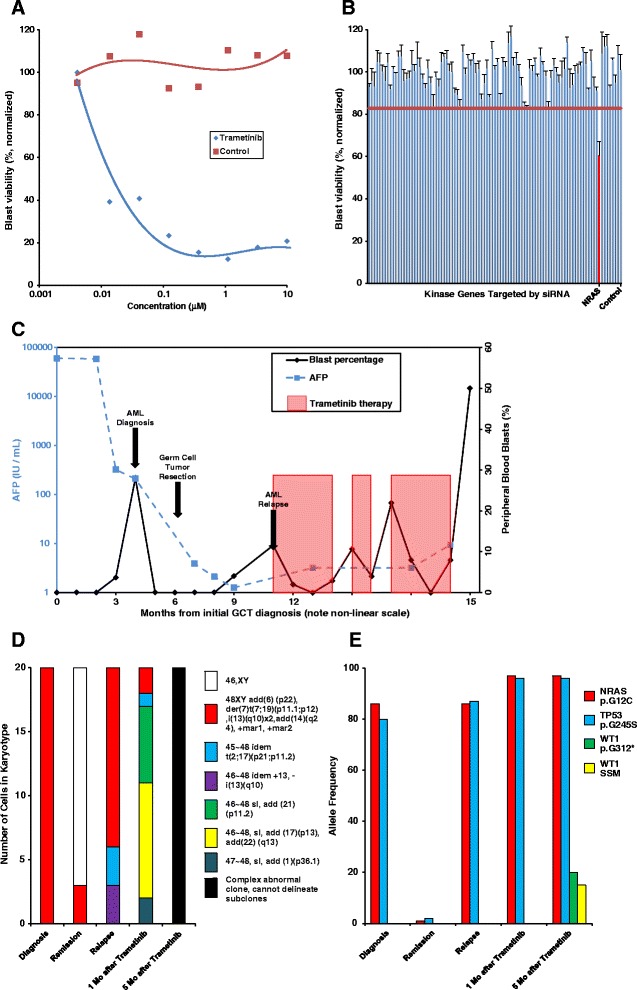
Trametinib inhibits leukemic blast viability in vitro and in vivo and also during cytogenetic and molecular clonal evolution. **a** Trametinib inhibits leukemic blast viability in vitro in a dose-dependent manner. **b** siRNA-mediated knock-down of NRAS decreases in vitro blast viability (*horizontal red bar* = 95 % percentile. **c** Trametinib therapy correlates with partial remission of leukemic blasts. Blast counts (*black line*) decreased when on trametinib therapy (*red shaded boxes*) following AML relapse after standard induction therapy. Note the non-linear time scale. **d** Karyotyping demonstrates clonal evolution before and during trametinib therapy. **e** Targeted massively parallel sequencing demonstrates persistent *NRAS* and *TP53* missense point mutations before and during trametinib therapy; WT1 mutations were identified at the time of germ cell tumor relapse. Identical NRAS and TP53 mutations were identified by targeted sequencing analysis of the patient’s mediastinal germ cell tumor

A 23-year-old male presented with fevers, hemoptysis, and an 18-cm anterior mediastinal mass (Fig. 1a). A computed tomography-guided core biopsy of the mass contained mostly necrotic tissue with rare atypical cells that were partially positive for alpha-fetoprotein (AFP), glypican, and pancytokeratin by immunohistochemistry. Serum AFP was elevated at >60,000 ng/mL, leading to a presumptive diagnosis of a mediastinal germ cell tumor. The patient was treated with four cycles of VIP (vinblastine, ifosfamide, etoposide) with concomitant decrease in serum AFP to 300 ng/mL. Prior to resection of the residual mediastinal mass (4 months after the original diagnosis), a complete blood count (CBC) demonstrated elevation of the WBC to 55 k/μL with 28 % circulating myeloid blasts. His lactate dehydrogenase (LDH) was markedly elevated at 17,481 U/L, although his AFP decreased to 211 ng/mL. A bone marrow biopsy demonstrated acute erythroid leukemia with 70–80 % markedly left-shifted erythroid precursors with dysplastic and megaloblastoid changes and myeloid blasts accounting for more than 20 % of non-erythroid cell population (Fig. 1b). Cytogenetics showed a complex clone (Fig. 2d). Targeted massively parallel sequencing analysis also identified *TP53* p.G245S and *NRAS* p.G12C point mutations at 87 and 86 % allele frequency, respectively (see Additional file 1 and Additional file 2: Table S1 for details). As the patient’s mediastinal mass had not yet been resected, a regimen that would treat both his AML and his residual germ cell tumor (containing platinum-based therapy) was desired. For this reason, he was treated with HAEP (high-dose cytarabine, etoposide, and cisplatin), previously reported as an effective salvage therapy for AML [11]. Following induction, the patient achieved morphologic complete remission (CR) of his AML; however, low-level residual disease was identified by cytogenetics (3 of 20 cells with the prior abnormal clone) and massively parallel sequencing analysis (1 % TP53 and 2 % NRAS mutant allele frequencies).

He was treated with a single cycle of HAEP consolidation therapy, after which he underwent resection of the mediastinal mass. Histopathologic examination demonstrated mature teratoma with foci of rhabdomyosarcoma and angiosarcoma (Fig. 1c, d). Targeted Sanger-based DNA sequencing analysis identified the same *TP53* and *NRAS* point mutations present in the acute erythroid leukemia, confirming a shared clonal origin.

Six weeks after resection of the mediastinal mass, a restaging bone marrow biopsy demonstrated relapsed disease with a morphologically abnormal erythroid series, 5–10 % myeloid blasts, and persistent *TP53* and *NRAS* mutations at 87 and 86 % allele frequency (Fig. 2e). Cytogenetics identified the original abnormal clone and the emergence of two additional clones (Fig. 2d). Since the patient was not fit for re-induction chemotherapy due to recent surgery, his leukemic cells were isolated and tested against a panel of small molecule kinase inhibitors to identify in vitro drug sensitivity. Trametinib was identified as one of the most potent in vitro inhibitors of leukemic cell viability (Fig. 2a). In vitro small inhibitory RNA (siRNA) studies further identified that silencing of NRAS had a profound inhibitory effect on blast viability (Fig. 2b), consistent with his known activating NRAS mutation (for full list of silenced genes please see Additional file 3: Table S2). The patient began treatment with single-agent trametinib therapy obtained on a compassionate use basis and had an immediate reduction in his peripheral blasts after a week of therapy (Fig. 2c).

A bone marrow biopsy after 1 month of trametinib revealed a slight reduction in the blast count (4–5 %) but persistent erythroid dysplasia. Although the AML remained stable, multiple cytogenetically complex clones were identified and the TP53 and NRAS mutant allele frequency increased to 96 and 97 %, respectively (Fig. 2e). After 3 months of trametinib monotherapy, the patient developed pneumonia and *Staphylococcus aureus* bacteremia. A bone marrow biopsy at that time demonstrated 8 % blasts and erythroid dysplasia. Trametinib was held twice due to infections, and during these intervals, there was an increase in peripheral blasts, which resolved following resumption of trametinib (Fig. 2c). A bone marrow biopsy after 5 months of trametinib therapy demonstrated stability of his AML with 6 % blasts and erythroid dysplasia. Karyotyping and repeat sequencing analysis demonstrated continued clonal evolution, with multiple cytogenetically complex subclones (Fig. 2d). There was a persistent high-level NRAS and TP53 mutant allele frequency but two additional WT1 point mutations were also identified at allele frequencies of 20 and 15 %, further indicating clonal evolution (Fig. 2e). Repeat testing confirmed that the leukemic clone remained sensitive to trametinib in vitro (data not shown). However, imaging studies performed in preparation for allogeneic stem cell transplantation demonstrated a 3-cm pulmonary nodule and serum AFP also rose to 12 ng/dL, consistent with relapse of metastatic GCT. Given his poor prognosis, the patient opted for palliative care and died a month later with frank relapse of his AML after trametinib discontinuation.

### Discussion

Although exceedingly rare, at least 60 cases of concurrent mediastinal GCT and hematologic malignancy have been reported in the literature. A common clonal origin was long suspected given the temporal relationship between the two malignancies, and recent work has convincingly demonstrated conserved isochromosome 12p rearrangements as well as somatic point mutations [12]. We provide further evidence of the clonal relationship between GCT and acute erythroid leukemia based on shared *NRAS* and *TP53* mutations. Furthermore, we demonstrate that targeted sequencing in combination with functional screening can identify active drugs for these rare diseases.

Massively parallel sequencing has identified a number of recurrent mutations in AML. However, the heterogeneity of mutations [13] precludes a uniform approach to treatment. Furthermore, the functional relevance of identified mutations is not always known for each AML patient. In light of this, our group has developed a tripartite approach to identifying therapeutic targets through (1) small molecule inhibitor screens, (2) siRNA knock-down, and (3) targeted massively parallel sequencing. In the presented case, an activating *NRAS* mutation and a TP53 mutation were identified by sequencing. Parallel siRNA and small molecule screens demonstrated the functional importance of the activated NRAS/Raf/MEK/ERK pathway in vitro. MEK inhibitors such as trametinib have shown activity in RAS-mutated AML; however, the overall response rate is only 28 %, indicating that a RAS mutation by itself is not sufficient to predict which patients will respond to therapy [14]. While trametinib monotherapy did not lead to CR in this patient, it did reduce peripheral blast counts and provided 6 months of disease control, allowing time to potentially coordinate a stem cell transplant. This response is consistent with pre-clinical models of RAS-activated AML in which trametinib inhibited leukemic proliferation but did not eradicate disease [15].

Disease control by trametinib also highlighted another interesting aspect of this case. Progressive clonal evolution was identified both through increasing cytogenetic complexity with development of at least four subclones. Targeted massively parallel sequencing analysis also demonstrated clonal evolution, with the development of two point mutations in WT1. We hypothesize that the p53 mutation contributed to the complex clonal evolution and disease progression, as loss of p53 function is known to impair DNA repair and genomic stability [16].

The use of next-generation sequencing to identify potentially targetable mutations is an emerging area of interest, particularly for rare or refractory malignancies. A small clinical series using sunitinib to treat refractory GCT identified amplification of the RET gene in one exceptional responder [17]. Other potential approaches to treating rare malignancies include basket trials, in which patients with a specified mutations are treated with targeted therapy irrespective of their underlying malignancy, or umbrella trials, in which patients with a defined cancers type are subclassified by genotype, and then treated based on their molecular classification [18]. Our approach blends genotype with functional in vitro drug screen as an additional predictor of targeted drug response to identify potential therapeutic targets.

## Conclusions

In conclusion, we report a case of metasynchronous mediastinal GCT and AML in a young male patient with identical TP53 and NRAS mutations in both malignancies. In vitro functional assays were used to confirm the importance of the NRAS mutation to leukemic blast proliferation and the ability of trametinib to inhibit growth. Trametinib therapy led to sustained disease control in vivo over 6 months following failure of conventional cytotoxic chemotherapy. This case highlights the power of in vitro functional assays to identify novel targeted agents to treat rare and aggressive malignancies and supports the exploratory use of small molecule kinase inhibitors in the treatment of AML, particularly in rare presentations of AML where therapeutic options are limited and outcomes are poor.

### Consents

Written informed consent was obtained from the patient to participate in a trial which would allow collection and sequencing of his leukemic blasts for sequencing and testing against a panel of small molecule inhibitors. In addition, he consented to sequencing of his normal tissue and his germ cell tumor. The patient is deceased and had no next of kin or legal guardian, so consent for publication of this case report and any accompanying images was not obtained. This lack of support contributed to his inability to proceed to allogeneic stem cell transplant.

## Additional files

Additional file 1: Supplemental information on the methods of the next-generation sequencing panel at OHSU used for analysis. (DOC 29.5 kb) Additional file 2: Table S1. Table of genes tested by the next-generation sequencing panel. (PPTX 58.1 kb) Additional file 3: Table S2. Table of genes targeted by siRNA panel in Fig. 2. (PPTX 47.3 kb)

## Abbreviations

AFP: alpha-fetoprotein
AML: acute myeloid leukemia
CBC: complete blood count
CR: complete remission
GCT: germ cell tumor
LDH: lactate dehydrogenase
siRNA: small inhibitory RNA
