# Supplementary material for: Functional and genetic screening of acute myeloid leukemia associated with mediastinal germ cell tumor identifies MEK inhibitor as an active clinical agent
Source: J Hematol Oncol. 2016 Mar 31;9:31. doi: 10.1186/s13045-016-0258-1 (PMC4815159; doi:10.1186/s13045-016-0258-1)
Supplement: Additional file 1: — Supplemental information on the methods of the next-generation sequencing panel at OHSU used for analysis. (DOC 29.5 kb) [file 13045_2016_258_MOESM1_ESM.doc]

**SUPPLEMENTAL INFORMATION**

**MATERIALS AND METHODS**

All laboratory, pathologic and genetic testing was performed in the context of standard clinical care. All protocols were approved by the Oregon Health & Science University institutional review board.

***Immunohistochemical staining:*** Standard methods were employed with an automated Ventana (Tucson, AZ) staining system with Ultraview (Ventana) detection chemistry.

***Sequencing analysis:*** Targeted massively parallel sequencing analysis was performed on a fresh bone marrow aspirate sample. A 42 gene panel (Additional file 3: Table S2) was designed with the highly multiplexed Ion AmpliSeq Designer (Life Technologies) software.  The AmpliSeq amplicon libraries were prepared according to the method described previously^1^. A total of 20ng of DNA was used for analysis. After adapters/IonXpress barcode ligation, the concentration of the AmpliSeq library was assessed using the Ion Library Quantitation Kit (Life Technologies, CA). Sequencing was performed on a PGM sequencer (Life Technologies, CA) using the Ion PGM^TM^ 200 Sequencing kit according to the manufacturer’s protocol.  Variants were identified with Variant Caller software version 3.2.1 using customized parameters.  Raw sequence reads were visualized and investigated with Integrative Genomics Viewer (Broad Institute, [http://www.broadinstitute.org/igv/](https://mail.ohsu.edu/owa/redir.aspx?SURL=HQnyhB2Gk6VVFVrThwkfEveqcMHyOOKNNSpWS1XVtTH0jN_7nGfSCGgAdAB0AHAAOgAvAC8AdwB3AHcALgBiAHIAbwBhAGQAaQBuAHMAdABpAHQAdQB0AGUALgBvAHIAZwAvAGkAZwB2AC8A&URL=http%3A%2F%2Fwww.broadinstitute.org%2Figv%2F)).  A lab-developed algorithm was used for variant annotation against public databases such as COSMIC (http://cancer.sanger.ac.uk/cancergenome/projects/cosmic/).  Standard Sanger-based DNA sequencing of NRAS and TP53 was performed on formalin fixed paraffin embedded tissue from the mediastinal germ cell tumor.

***Inhibitor panels and siRNA studies:*** Blasts were collected from bone marrow aspirate material, isolated, and subjected to a panel of kinase inhibitors and small inhibitory RNA (siRNA) molecules as previously described^2,3^.

**SUPPLEMENTAL REFERENCES**

1. Beadling C, Neff TL, Heinrich MC, Rhodes K, Thornton M, Leamon J, Andersen M, Corless CL. *Combining highly multiplexed PCR with semiconductor-based sequencing for rapid cancer genotyping.* J Mol Diagn. 2013 Mar;**15**(2):171-6.

2. Tyner JW, Yang WF, Bankhead A 3rd, Fan G, Fletcher LB, Bryant J, Glover JM, Chang BH, Spurgeon SE, Fleming WH, Kovacsovics T, Gotlib JR, Oh ST, Deininger MW, Zwaan CM, Den Boer ML, van den Heuvel-Eibrink MM, O'Hare T, Druker BJ, Loriaux MM. *Kinase pathway dependence in primary human leukemias determined by rapid inhibitor screening.* Cancer Res. 2013 Jan 1;**73**(1):285-96.

3. Tyner JW, Deininger MW, Loriaux MM, Chang BH, Gotlib JR, Willis SG, Erickson H, Kovacsovics T, O'Hare T, Heinrich MC, Druker BJ. *RNAi screen for rapid therapeutic target identification in leukemia patients.* Proc Natl Acad Sci U S A. 2009 May 26;**106**(21):8695-700.
