# Supplementary material for: Functional and genetic screening of acute myeloid leukemia associated with mediastinal germ cell tumor identifies MEK inhibitor as an active clinical agent
Source: J Hematol Oncol. 2016 Mar 31;9:31. doi: 10.1186/s13045-016-0258-1 (PMC4815159; doi:10.1186/s13045-016-0258-1)
Supplement: Additional file 2: Table S1. — Table of genes tested by the next-generation sequencing panel. (PPTX 58.1 kb) [file 13045_2016_258_MOESM2_ESM.pptx]

## Slide 1
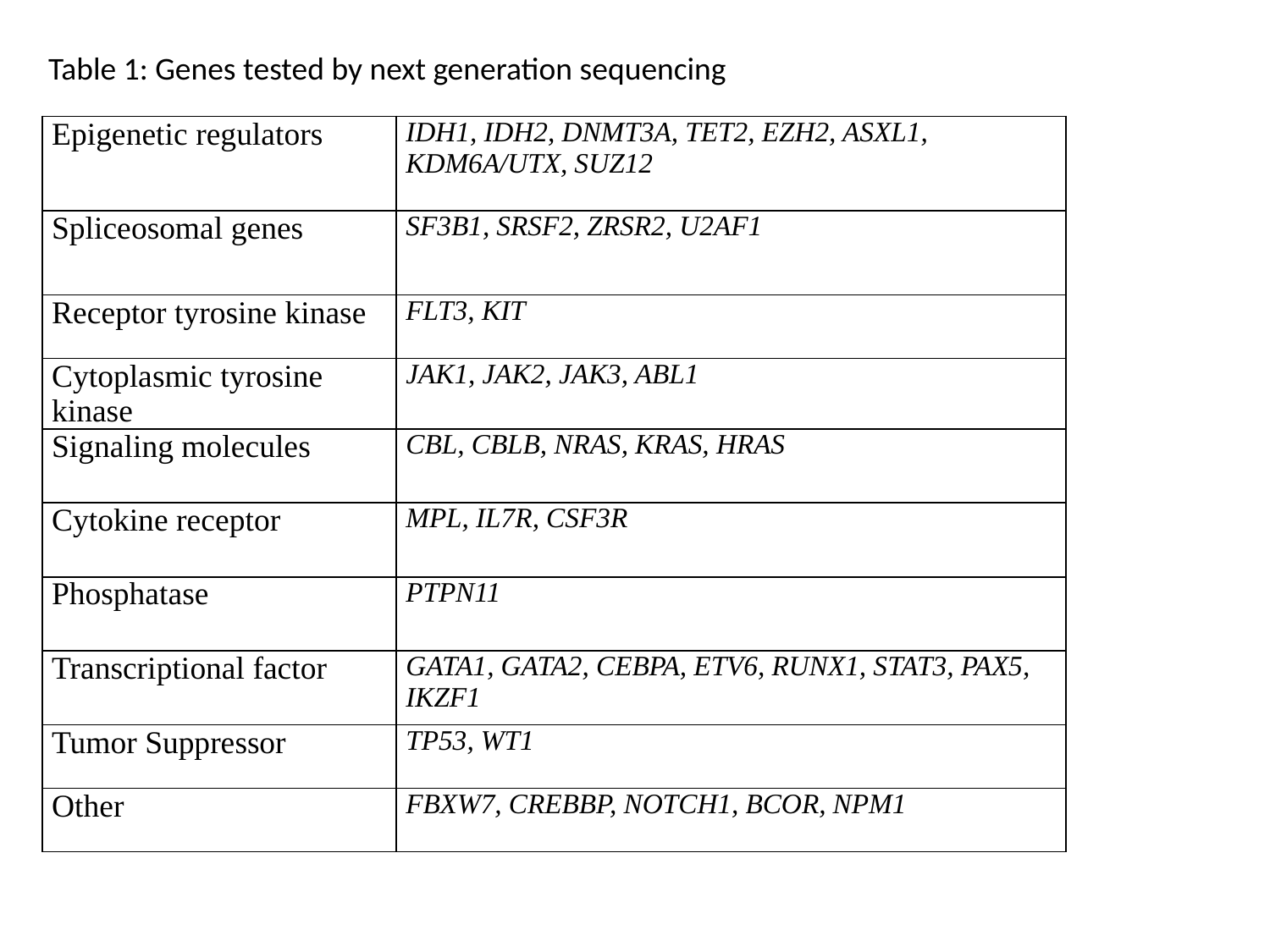

Table 1: Genes tested by next generation sequencing
| Epigenetic regulators | IDH1, IDH2, DNMT3A, TET2, EZH2, ASXL1, KDM6A/UTX, SUZ12 |
| --- | --- |
| Spliceosomal genes | SF3B1, SRSF2, ZRSR2, U2AF1 |
| Receptor tyrosine kinase | FLT3, KIT |
| Cytoplasmic tyrosine kinase | JAK1, JAK2, JAK3, ABL1 |
| Signaling molecules | CBL, CBLB, NRAS, KRAS, HRAS |
| Cytokine receptor | MPL, IL7R, CSF3R |
| Phosphatase | PTPN11 |
| Transcriptional factor | GATA1, GATA2, CEBPA, ETV6, RUNX1, STAT3, PAX5, IKZF1 |
| Tumor Suppressor | TP53, WT1 |
| Other | FBXW7, CREBBP, NOTCH1, BCOR, NPM1 |
