# Supplementary material for: Functional and genetic screening of acute myeloid leukemia associated with mediastinal germ cell tumor identifies MEK inhibitor as an active clinical agent
Source: J Hematol Oncol. 2016 Mar 31;9:31. doi: 10.1186/s13045-016-0258-1 (PMC4815159; doi:10.1186/s13045-016-0258-1)
Supplement: Additional file 3: Table S2. — Table of genes targeted by siRNA panel in Fig. 2. (PPTX 47.3 kb) [file 13045_2016_258_MOESM3_ESM.pptx]

## Slide 1
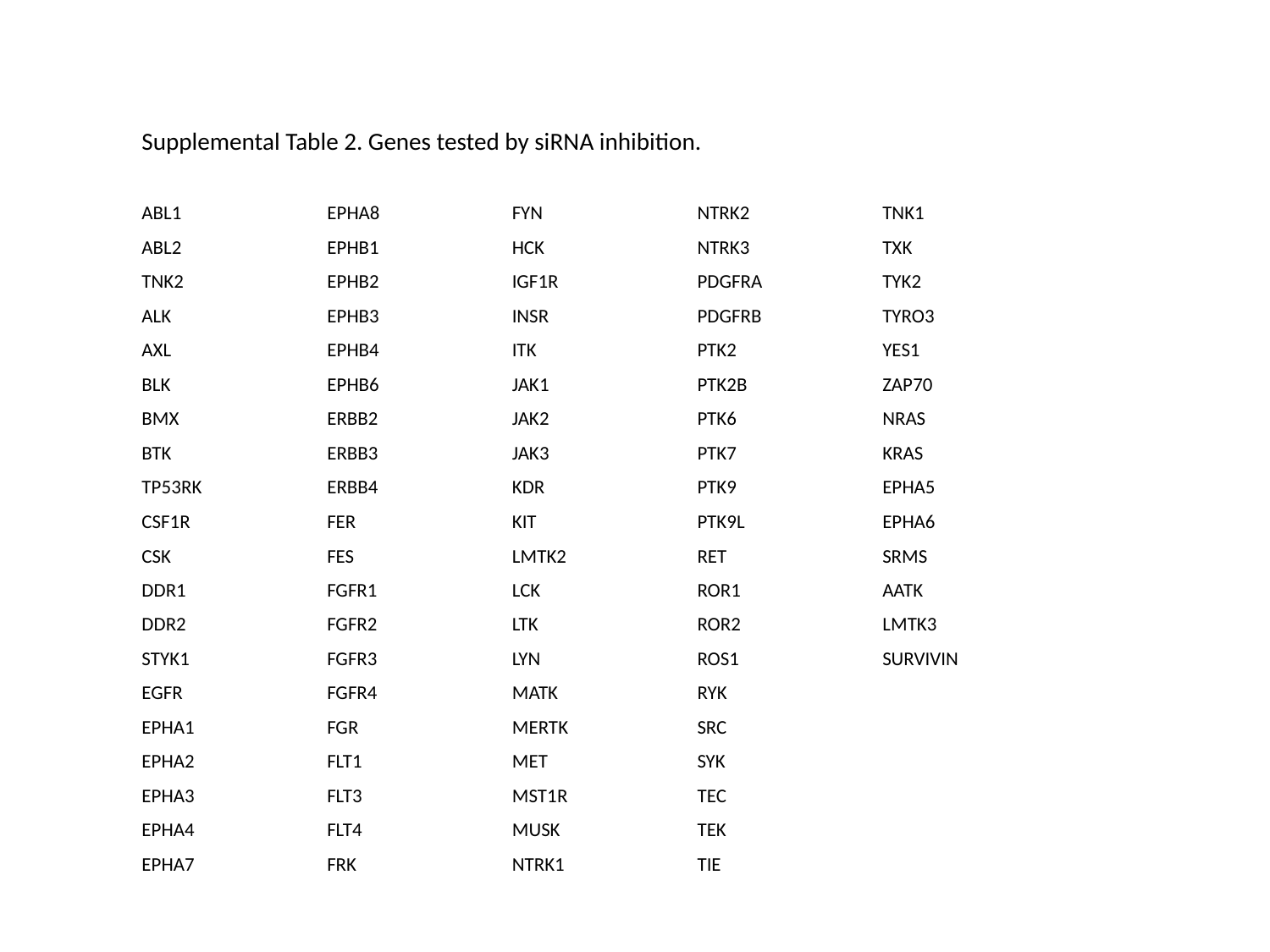

| Supplemental Table 2. Genes tested by siRNA inhibition. | | | | |
| --- | --- | --- | --- | --- |
| | | | | |
| ABL1 | EPHA8 | FYN | NTRK2 | TNK1 |
| ABL2 | EPHB1 | HCK | NTRK3 | TXK |
| TNK2 | EPHB2 | IGF1R | PDGFRA | TYK2 |
| ALK | EPHB3 | INSR | PDGFRB | TYRO3 |
| AXL | EPHB4 | ITK | PTK2 | YES1 |
| BLK | EPHB6 | JAK1 | PTK2B | ZAP70 |
| BMX | ERBB2 | JAK2 | PTK6 | NRAS |
| BTK | ERBB3 | JAK3 | PTK7 | KRAS |
| TP53RK | ERBB4 | KDR | PTK9 | EPHA5 |
| CSF1R | FER | KIT | PTK9L | EPHA6 |
| CSK | FES | LMTK2 | RET | SRMS |
| DDR1 | FGFR1 | LCK | ROR1 | AATK |
| DDR2 | FGFR2 | LTK | ROR2 | LMTK3 |
| STYK1 | FGFR3 | LYN | ROS1 | SURVIVIN |
| EGFR | FGFR4 | MATK | RYK | |
| EPHA1 | FGR | MERTK | SRC | |
| EPHA2 | FLT1 | MET | SYK | |
| EPHA3 | FLT3 | MST1R | TEC | |
| EPHA4 | FLT4 | MUSK | TEK | |
| EPHA7 | FRK | NTRK1 | TIE | |
